# Diverse Action of Selected Statins on Skeletal Muscle Cells—An Attempt to Explain the Protective Effect of Geranylgeraniol (GGOH) in Statin-Associated Myopathy (SAM)

**DOI:** 10.3390/jcm8050694

**Published:** 2019-05-16

**Authors:** Anna Jaśkiewicz, Beata Pająk, Magdalena Łabieniec-Watała, Clara De Palma, Arkadiusz Orzechowski

**Affiliations:** 1 Department of Physiological Sciences, Faculty of Veterinary Medicine, Warsaw University of Life Sciences—SGGW, Warsaw, 02-787 Mazowieckie, Poland; ancpatrin@gmail.com; 2 Independent Laboratory of Genetics and Molecular Biology, The General Karol Kaczkowski Military Institute of Hygiene and Epidemiology, Warsaw, 01-163 Mazowieckie, Poland; bepaj@wp.pl; 3 Faculty of Biology and Environmental Protection, University of Łódź, 90-236 Łódzkie, Poland; magdalena.labieniec@biol.uni.lodz.pl; 4 Department of Biomedical and Clinical Sciences “Luigi Sacco”, Università degli Studi di Milano, 20157 Milano, Italy; 5 Unit of Clinical Pharmacology, University Hospital “Luigi Sacco”-ASST Fatebenefratelli Sacco, 20157 Milano, Italy

**Keywords:** skeletal muscle cell viability, statins, myotoxicity, geranylgeraniol, water-soluble cholesterol, statin-associated myopathy, mitochondrial bioenergetics

## Abstract

The present study is centered on molecular mechanisms of the cytoprotective effect of geranylgeraniol (GGOH) in skeletal muscle harmed by statin-associated myopathy (SAM). GGOH via autophagy induction was purportedly assumed to prevent skeletal muscle viability impaired by statins, atorvastatin (ATR) or simvastatin (SIM). The C2C12 cell line was used as the ‘in vitro’ model of muscle cells at different stages of muscle formation, and the effect of ATR or SIM on the cell viability, protein expression and mitochondrial respiration were tested. Autophagy seems to be important for the differentiation of muscle cells; however, it did not participate in the observed GGOH cytoprotective effects. We showed that ATR- and SIM-dependent loss in cell viability was reversed by GGOH co-treatment, although GGOH did not reverse the ATR-induced drop in the cytochrome *c* oxidase protein expression level. It has been unambiguously revealed that the mitochondria of C2C12 cells are not sensitive to SIM, although ATR effectively inhibits mitochondrial respiration. GGOH restored proper mitochondria functioning. Apoptosis might, to some extent, explain the lower viability of statin-treated myotubes as the pan-caspase inhibitor, N-Benzyloxycarbonyl-Val-Ala-Asp(O-Me) fluoromethyl ketone (Z-VAD-FMK), partly reversed ATR- or SIM-induced cytotoxic effects; however, it does not do so in conjunction with caspase-3. It appears that the calpain inhibitor, N-Acetyl-L-leucyl-L-leucyl-L-norleucinal (ALLM), restored the viability that was reduced by ATR and SIM (*p* **<** 0.001). GGOH prevents SAM, in part, as a consequence of a caspase-3 independent pathway, probably by calpain system inactivation.

## 1. Introduction

Statins are widely used cholesterol-lowering drugs, with proven protective cardiovascular activity [1]. However, the administration of these 3-hydroxy-3-methylglutaryl coenzyme A reductase inhibitors (HMGCR; EC 1.1.1.88) [2] may lead to adverse effects such as statin intolerance associated with statin-associated myopathy (SAM) [3]. Reported symptoms of SAM include myalgia, fatigue, weakness, low back or proximal muscle pain, and occasionally, fatal rhabdomyolysis [4]. Although the incidence of statin-induced side effects based on clinical trials is fairly low (195 cases per 100,000 patient-years), it can be underestimated as patients with increased risk of SAM are often excluded from studies [5]. A prospective observational follow-up study, known as Prediction of Muscular Risk and Observational Conditions (PRIMO), revealed that 11% of hyperlipidemic patients subjected to a high-dose statin therapy reported muscle symptoms within a 12-month period [6]. A higher incidence was observed in heavily exercised muscles [7]. Nonetheless, since the frequency of myalgia in statin-treated subjects was found to be almost indistinguishable regardless of atorvastatin dose, it is very unlikely that SAM is connected with statin dose or low-density lipoprotein (LDL) levels [8,9]. In the search for a possible explanation of SAM, several hypotheses have been formulated [10], such as (i) low cholesterol in sarcolemma, underpinning mechanical injury during muscle contractions; (ii) low coenzyme Q10 (ubiquinone) and impaired mitochondrial function due to the reduced stores of this electron carrier; (iii) reduced N-linked glycosylation (NLG) of proteins owing to diminished dolichol synthesis; and (iiii) dropped formation of non-sterol isoprenoids that are essential for prenylation of cellular proteins with GTP-binding proteins that control cytoskeleton organization and intracellular vesicular transport. Theoretically, all of the above could result from statin-dependent impairment of the mevalonate pathway [10,11,12,13], even though experimental data do not support every case. Cholesterol does not seem to be critical for SAM as the inhibition of cholesterol synthesis at the level of squalene synthase does not induce myotoxicity [14]. Moreover, this observation was further confirmed by other authors who blocked terminal steps in sterol formation without any noticeable SAM [15,16]. It was apparent that reduced synthesis of coenzyme Q (ubiquinone) [17,18] was also not an important factor in muscle vulnerability to statins [12,19] as a statin-dependent fall in serum ubiquinone concentration does not lessen muscle levels of this isoprenoid [20].

Numerous studies reported a statin-dependent deficit in mitochondrial oxidative phosphorylation with SAM in specific preclinical animal and cellular models [21,22,23,24,25,26,27], although debate still continues as to whether or not, and how, mitochondria depletion contributes to the pathogenesis of statin myopathy [21,28].

Dolichol belongs to isoprenoids of the mevalonate pathway that are involved in NLG, a co-translational covalent protein modification [29]. Given that NLG is vital for some important plasma membrane proteins, such as components of the DGC or IGF-1 receptors, one may expect that SAM is caused by the deficit of dolichol [30,31]. Although statins may impair NLG, the precise position of the process in SAM could be indirectly assessed from the examination of the effect of dolichol (DOH) on muscle cell survival. 

Most evidence points to protein prenylation with farnesol (FOH) and/or geranylgeraniol (GGOH) as fundamental to muscle cell viability, growth and differentiation [10,32]. Several very important proteins (lamins A/C, selenoprotein N, α- and β-dystroglycan, GTP-binding proteins) need prenylation for optimal function. In view of this, statins were no longer capable of inducing apoptosis in muscle cells once the protein geranylgeranylation was restored by mevalonate, farnesol or geranylgeraniol [19]. In addition, ubiquinone could not rescue muscle cells from SAM [12]. Isoprenoids are fundamental for prenylation of small guanosine triphosphate (GTP)-binding proteins (G-proteins, Ras (including Rap), Rac, and Rho). The levels of prenylated Rap1 demonstrated a statin-induced fall in muscle cell viability; however, it was reestablished by the addition of GGOH [33]. Occasionally, the so-called “salvage pathway” (GGOH is utilized for isoprenylation, and FOH for sterol biosynthesis and isoprenylation) maintains isoprenylation that is feasible even when the mevalonate pathway is destroyed [34]. Additionally, the decline in protein prenylation led to elevated cytoplasmic Ca^2+^ levels that were most likely released from mitochondria and sarcoplasmic reticulum (SE), [24]. Ca^2+^ flux from SE is correlated with damages of ryanodine receptors (RyRs), and the overexpression of the RyR3 subtype is suggestive of the intracellular calcium leak not linked to elevated serum creatine kinase (CK), [35]. Calcium entry disturbs normal intracellular homeostasis and may trigger activity of proteases such as caspases and calpains [36,37].

In the present study, we chose atorvastatin (ATR) as the less lipophilic and simvastatin (SIM) as the more lipophilic statin in order to distinguish their relative myotoxicity. In this regard, it is noteworthy that myotoxicity determined by the elevated serum levels of CK or impaired mitochondrial respiration is higher for less lipophilic statins [21]. We hypothesized that SAM is caused by muscle cell apoptosis whereas the cytoprotective effect of GGOH could be attributable to autophagy induction as beforehand we reported an elevated level of Beclin-1 in response to GGOH [33]. To check if cell loss was due to caspase and/or calpain activation, together with statins, we administered the pan-caspase inhibitor N-Benzyloxycarbonyl-Val-Ala-Asp(O-Me) fluoromethyl ketone (Z-VAD-FMK) vs. the calpain inhibitor N-Acetyl-L-leucyl-L-leucyl-L-norleucinal (ALLM). To examine whether GGOH accelerates autophagy, we conducted autophagy fluxes with the use of chloroquine (CQ). Indices of either cell death or cell survival together with immunoblotting were selected as molecular biology markers.

## 2. Experimental Section

### 2.1. Reagents and Antibodies

Malate, glutamate, succinate, digitonin, rotenone, cytochrome *c*, FCCP (carbonyl cyanide p-trifluoromethoxyphenylhydrazone), antimycin A, ADP, trichloroacetic acid (TCA), acetic acid glacial (99.85%), ATR, SIM, all-trans-geranylgeraniol (GGOH), poly(ethylene-glycol 600)-Cholesterol conjugate (Chol-PEG), poly(ethylene-glycol 600) (PEG), (3-(4,5-dimethylthiazol-2-yl)-2-5-diphenyltetrazolium bromide) (MTT), and bovine serum albumin (BSA) were purchased from Sigma Aldrich, Saint Lois, MO, USA. Sulforhodamine B (75%, SRB) was purchased from Calbiochem, Merck Millipore (Darmstadt, Germany). MitoTracker Green FM fluorescent mitochondrial stain was purchased from Life Technologies/Thermo Fisher Scientific (Waltham, MA, USA). All the other reagents and solvents that were used in this study were of the highest analytical reagent grade. 

### 2.2. Cell Cultures and Treatments

The murine skeletal muscle cell line, C2C12, was obtained from the European Collection of Animal Cell Cultures (ECAAC, Salisbury, UK). Cells were initially cultured in growth media (GM) constituted by Dulbecco’s Modified Eagle’s Medium (DMEM, Biowest, Nuaillé, France) supplemented with 10% (v/v) heat-inactivated fetal bovine serum (FBS, Gibco, Carlsbad, CA, USA), Penicillin/Streptomycin (Life Technologies/Thermo Fisher Scientific, Waltham, MA, USA; 50 IU/mL/50 μg/mL), Gentamicin sulfate (Sigma Aldrich, Saint Lois, MO, USA; 20 μg/mL), and Fungizone/Amphotericin B (Thermo Fisher Scientific, Waltham, MA, USA; 1 μg/mL), and grown until 70–80% confluence. Spontaneous differentiation by growth factor withdrawal was induced, replacing growth medium with a differentiation medium (DM) consisting of DMEM supplemented with 2% (v/v) heat-inactivated horse serum (HS, Gibco, Carlsbad, CA, USA) and the same antibiotic/antimycotic cocktail as the GM, then being further incubated for up to 5 days. Muscle cells and syncytia were harvested on day 1, 3, and 5 of myogenic differentiation time points at which they displayed different phenotypes (Figure S1). At day 1, cells exhibited proliferating myoblasts, at day 3 differentiating myotubes, and at day 5 differentiated myotubes.

Treatment of the cultures with the different experimental factors tested in the current work was performed 24, 72, or 120 h prior to cell harvesting based on the respective half maximal inhibitory concentrations (IC_50_) for statins and at a neutral concentration (not different from untreated control) for other experimental factors. Statins (ATR or SIM) were administered at various concentrations during differentiation: ATR: Day 1—100 μM, Day 1–3—46 μM, Day 1–5—36 μM; SIM: Day 1—125 μM, Day 1–3—10 μM, Day 1–5—7.5 μM. Statin concentrations seem to be irrelevant regarding the plasma concentrations observed in mice and humans (nanomolar). The discrepancy is explained by the duration of the experiment, which lasted merely 5 days (in vivo myogenesis lasts considerably longer—at least few weeks). As reported by us, the IC50 concentration of statins is inversely proportional to the time of treatment. ATR, SIM, GGOH and Chol-PEG were administered throughout the duration of the experiment [33]. In contrast, ALLM, CQ, and Z-VAD were administered for 24 h at the selected day of myogenesis (day 1, 3 or 5) and prior to data collection.

### 2.3. Assessment of Cell Viability

Cell viability was assessed by evaluating the ability of cells to convert soluble MTT (3-(4,5-dimethylthiazol-2-yl)-2-5-diphenyltetrazolium bromide) into an insoluble purple formazan as described [38]. Briefly, cells grown and differentiated as above were incubated for 4 h at 37 °C with MTT (0.5 mg/mL in DMEM without phenol red). Water-insoluble formazan was immediately dissolved in DMSO (100 μL per well). In brief, the sulforhodamine B (SRB) assay was used for the measurement of cellular protein content. The method described here has been optimized for the toxicity screening of compounds to adherent cells in a 96-well format described by Orellana and Kasinski [39]. After an experiment was completed, cell monolayers were fixed with 10% (w/v) trichloroacetic acid and stained for 30 min, after which the excess dye was removed by gentle washing repeatedly with 1% (vol/vol) acetic acid. The protein-bound dye was dissolved in 10 mM Tris base solution (pH 10.5). Color determination in MTT assay was measured at 570 nm and at 510 nm in SRB assay using a microplate reader TECAN 200 multiplate reader (Tecan Group Ltd., Männedorf, Switzerland). 

### 2.4. Mitochondrial Bioenergetics Measurements

The parameters of mitochondrial bioenergetics in tested cell line C2C12 were determined using an oxygraph-2k Oroboros (Oroboros Instruments, Innsbruck, Austria). DatLab software (Oroboros Instruments) was used for data acquisition and analysis. All measurements were performed at 37 °C in MIRO6 medium (0.5 mM EGTA, 3 mM MgCl2, 60 mM K-lactobionate, 20 mM taurine, 10 mM KH2PO4 20 mM Hepes, 110 mM sucrose and 1 g/L bovine serum albumin fatty acid-free, 280 U/mL catalase (pH 7.1)) with continuous stirring at 750 rpm in the presence of mitochondrial substrates and inhibitors, according to the procedures that were described in detail in References [40,41] with some modifications. Briefly, the cells were resuspended in respiration buffer (MIR06), added to each chamber, and allowed to stabilize under so-called routine respiration. Digitonin-permeabilized cell analysis was employed to assess complexes I and II and the optimal concentration of digitonin (5 mg/mL) was evaluated in an independent set of experiments. Routine respiration was identified in MiR06. After the routine respiration was established, the substrate, inhibitor and uncoupler protocol were started and respiration driven by Complex I was evaluated by adding glutamate (10 mM) malate (5 mM) and ADP at a saturating concentration (5 mM), revealing the CI maximal oxidative phosphorylation capacity (OXPHOS or State 3). In all variants, the lack of a significant increase in respiration after the addition of cytochrome *c* confirmed the integrity of the outer mitochondrial membrane, providing a quality control of digitonin-permeabilized cells. A subsequent titration of succinate (10 mM) led to evaluation of state 3 by convergent electron flow, defined as the oxido-reduction reactions from both complexes I and II. The maximal electron flow that is not associated with OXPHOS respiration (electron transport system (ETC) state) was assessed by the stepwise (1–5 µL) titration of FCCP (1 mM). Uncoupled complex-II-linked respiration was achieved by adding rotenone (0.5 µM). Finally, the respiratory chain was inhibited by antimycin A (2.5 µM) to obtain the residual oxygen flux (ROX). ROX after antymycin addition was subtracted from the steady-state respiration values.

Respiration values were calculated as the decrease in the oxygen concentration with time measured in closed chambers and expressed per milligram of protein. The protein concentration in each sample was determined by the Bradford assay.

To determine the mitochondrial bioenergetics, the tested cells (*n* = 5) were subjected to the following experimental treatments: CTRL (control), ATR, ATR + GGOH, ATR + Chol-PEG, SIM, SIM + GGOH, SIM + Chol-PEG.

The exposure of cells to pure GGOH and pure Chol-PEG (*n* = 3) was also made in order to check the effect of these compounds on mitochondrial bioenergetics. The main aim of this experimental stage was to rule out the possibility that GGOH and Chol-PEG may disrupt the mitochondrial respiration and affect the final conclusions.

Finally, the following mitochondrial states were calculated:

Routine respiration—mitochondrial respiration in the absence of exogenously added substrates and inhibitors;

State CI (state 3/OXPHOS I) respiration—ADP-stimulated respiration, supported by Complex I;

State CI + CII respiration (OXPHOS I&II)—ADP-stimulated respiration, supported by Complex I and Complex II;

State E (ETS capacity)—A respiratory Electron Transport System capacity. This state was induced by FCCP addition;

State CII respiration (CII)—mitochondrial respiration after Complex I inhibition, in the presence of succinate addition.

### 2.5. Western Blot Analysis

Cells, after being subject to the different treatments described above, were lysed in radioimmunoprecipitation assay buffer (RIPA) buffer (10 mM Na_4_P_2_O_7_, 50 mM 4-(2-hydroxyethyl)piperazine-1-ethanesulfonic acid (HEPES), 150 mM NaCl, 1% triton-X, 0.1% dodecyl sodium sulfate (SDS),10 mM ethylenediaminetetraacetic acid (EDTA), 100 mM NaF, pH 7.4) containing protease inhibitor cocktail (Roche, Mannheim, Germany), insoluble material removed by centrifugation (10,000 g, 5 min), and protein levels quantitated with Bradford reagent (Bio-Rad, Hercules, CA, USA). Each protein extract was adjusted to a 2 μg/μL concentration with the addition of Laemmli sample buffer (4X concentrate, Bio-Rad, Hercules, CA, USA), heated for 5 min at 95 °C, and separated on precast polyacrylamide gradient gels (7.5–12%). After being transferred to 0.2 μm pore size polyvinylidene difluoride (PVDF) blotting membranes (Bio-Rad, Hercules, CA, USA), membranes were blocked in either 5% (w/v) nonfat dried milk or 1% (w/v) BSA (Sigma Aldrich) diluted in Tris-buffered saline containing 0.1% (v/v) Tween 20 (TBS-T) and probed overnight at 4 °C with the respective primary antibodies, as follows: microtubule-associated proteins 1B/2B light chains 3B (MAP LC3-Ib/MAP LC3-IIb), Cleaved Caspase-3 (Cell Signaling Technology, Danvers, MA, USA), anti-MTCO1 (Abcam, Cambridge, MA, USA), and actin (C-11, Santa Cruz Biotechnology, Dallas, CO, USA). Membranes were subsequently incubated with the respective HRP-linked anti-rabbit, anti-mouse IgG (Cell Signaling Technology, Danvers, MA, USA) or anti-goat (Cell Signaling Technology, Danvers, MA, USA) antibodies and developed by ECL (Pierce™ ECL Western Blotting Substrate, Thermo Fisher Scientific). Densitometric quantification of band intensities was performed using analysis software (Image Studio Lite Version 5.2.5), Odyssey CLx Imaging System (LI-COR Biotechnology, GmbH, Bad Homburg, Germany) and the open-source image processing package Fiji (ImageJ). Levels of Cleaved Caspase-3, anti-MTCO1, MAP LC3-Ib/MAP LC3-IIb, and actin were carried out, probing the cell lysates obtained at each selected step of myogenesis with the pertinent antibodies.

### 2.6. Statistical Analyses

Mitochondrial respiration—the statistical calculations were made with the use of STATISTICA.PL software (v.13.1). The measurements were performed at least in duplicate, *n* = 3–5. The sample size was estimated for type I and type II statistical errors of 0.05 and 0.8, respectively. The results were shown as the mean ± SD (standard deviation). The data were tested for normal distribution using the Shapiro–Wilk test and variance homogeneity was verified using the Brown–Forsythe test. Following this testing, the data with a normal distribution were analysed with the use of parametric tests. The statistical significance between homogenous groups was evaluated using one-way ANOVA. Finally, a post hoc Tukey’s test was used. The post hoc power of the used tests was checked for each parametric analysis. A statistical test power below 80% was considered as an invalid outcome, and constructive conclusions were not formulated.

Viability studies and densitometry analyses—One-way ANOVA test with Tukey’s multiple comparisons and/or two-way ANOVA test followed by Bonferroni’s multiple comparisons were employed to analyze the data with the GraphPad PrismTM version 5.0 software (GraphPad Software Inc., San Diego, CA, USA). 

The results of (Time (proliferating myoblasts, differentiating myotubes, differentiated myotubes)) and (Treatments: (ATR, CQ, ATR + CQ, GGOH, ATR + GGOH, ATR + GGOH + CQ, Chol-PEG, ATR + Chol-PEG, ATR + Chol-PEG, ATR + Chol-PEG + CQ, SIM, CQ, SIM + CQ, GGOH, SIM + GGOH, SIM + GGOH + CQ, Chol-PEG, SIM + Chol-PEG, SIM + Chol-PEG + CQ) ) were analyzed accordingly. Error bars = standard error of mean (S.E.M.), and * *p* < 0.05, ** *p* < 0.01, *** *p* < 0.001 for comparison between the means. Results are obtained by the means of three independent experiments.

The publication of this manuscript implicates that all materials, data, computer code, and protocols associated with the publication are available to readers.

## 3. Results

### 3.1. Magnitude of Autophagy Increases in Differentiating Skeletal Muscle Cells. Rescuing Skeletal Muscle Cells from Statin-Induced Myotoxicity by GGOH is Independent of Autophagy

**Figure 1:**
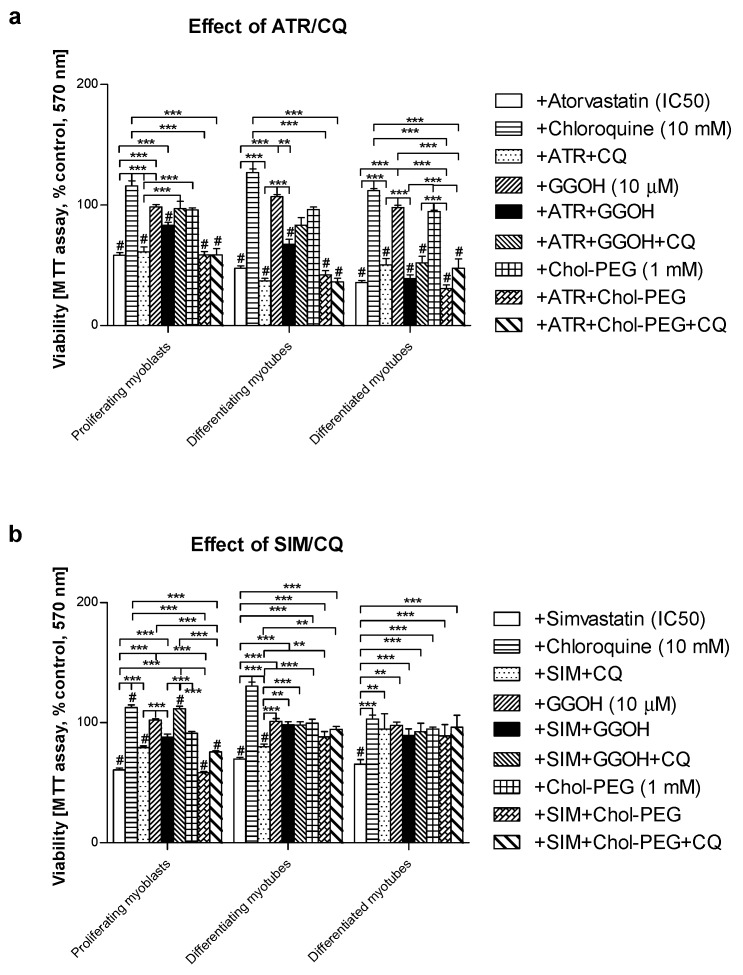
Effect of geranylgeraniol (GGOH, 10 µM) on cell viability (MTT assay) affected by mevalonate (MEV) pathway modulators (atorvastatin—ATR, simvastatin—SIM) or water-soluble cholesterol (Chol-PEG) in the presence or absence of chloroquine (CQ, 10 mM). Differentiating C2C12 myoblasts were exposed for 24, 72, or 120 h to statins (IC_50_) or water-soluble cholesterol (Chol-PEG), (Day 1—proliferating myoblasts; Day 3—differentiating myotubes; Day 5—differentiated myotubes). (**a**) ATR diminished fraction of viable cells (IC_50_). Neither CQ nor Chol-PEG or GGOH affected cell viability in comparison to control untreated cells (*p* > 0.05); GGOH but not Chol-PEG improved cell viability that had been reduced by ATR in proliferating myoblasts (*p* < 0.001). The effect of GGOH was not affected by CQ administration (*p* > 0.05); (**b**) SIM diminished the fraction of viable cells (IC_50_). Neither CQ nor water-soluble cholesterol (Chol-PEG) affected cell viability in comparison to control untreated cells (*p* > 0.05); GGOH but not Chol-PEG recovered cell viability reduced by SIM in proliferating myoblasts, differentiating and differentiated myotubes (*p* < 0.001). The effect of GGOH was not affected by CQ administration (*p* > 0.05); ** *p* < 0.01, *** *p* < 0.001, for comparison between the means. Statistically significant differences from untreated control cells are marked by # (at least at the level of *p* < 0.05). The results are indicative of three independent experiments performed in eight replicates.

Mouse C2C12 skeletal muscle cells were subjected to a 5-day differentiation program with or without co-treatment(s). In the previous report, the cell viability was retained by co-treatment with GGOH with a concomitant increase in the Beclin-1 protein expression levels, suggesting the contribution of autophagy to survival signal [33]. Autophagic flux was used to check whether the degradation phase inhibited by CQ affects muscle cell viability and protein synthesis. This study demonstrated that CQ itself is neutral to muscle cells; furthermore, its application together with statins did not affect statin-dependent loss in cell viability (Figure 1a,b). GGOH itself did not affect cell viability, but it considerably improved cell survival that had been worsened by statins (except for ATR) in differentiated myotubes (Figure 1a,b). CQ addition did not control GGOH-dependent cytoprotective effect (Figure 1a,b).

**Figure 2:**
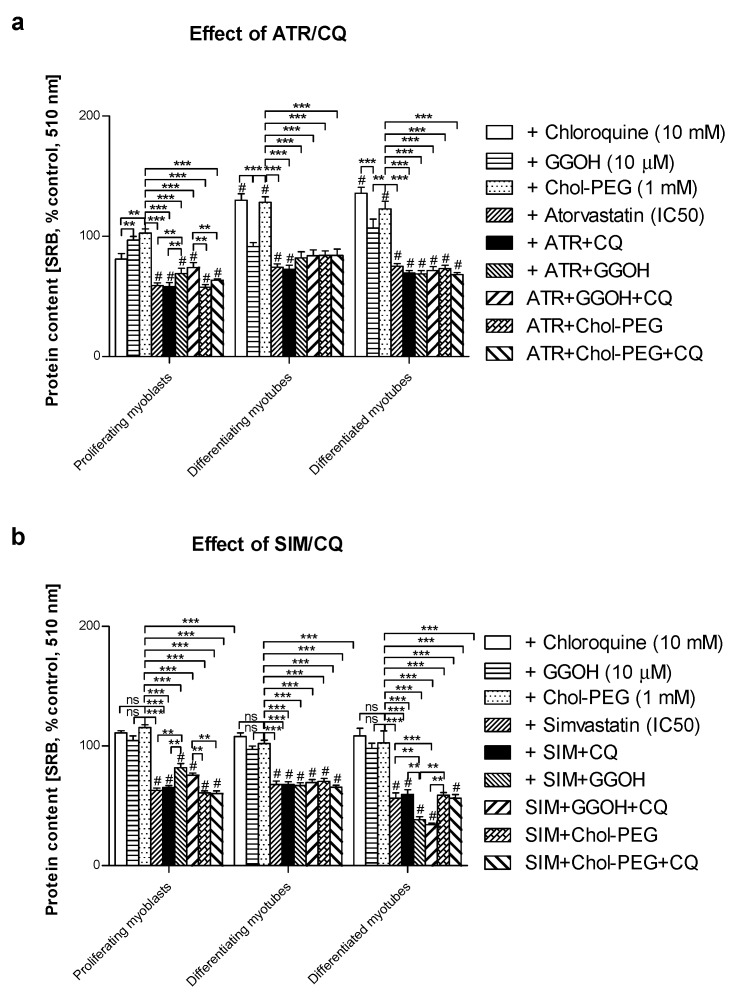
Effect of geranylgeraniol (GGOH, µ10 M) on the protein content (sulforhodamine B (SRB) incorporation) affected by MEV pathway modulators (atorvastatin—ATR, simvastatin—SIM) or water-soluble cholesterol (Chol-PEG) in the presence or absence of chloroquine (CQ, 10 mM). Differentiating C2C12 myoblasts were exposed for 24, 72 or 120 h to statins (IC_50_) or water-soluble cholesterol (Chol-PEG), (Day 1—proliferating myoblasts; Day 3—differentiating myotubes; Day 5—differentiated myotubes). (**a**) ATR diminished the protein content in viable cells (IC_50_). Neither CQ nor Chol-PEG or GGOH affected the protein content in comparison to control untreated cells (*p* > 0.05); GGOH but not Chol-PEG recovered cellular protein content reduced by ATR in proliferating myoblasts (*p* < 0.001). The effect of GGOH was not affected by CQ administration (*p* > 0.05); (**b**) SIM diminished the protein content in viable cells (IC_50_). Neither CQ nor Chol-PEG or GGOH affected the protein content in comparison to control untreated cells (*p* > 0.05); GGOH but not Chol-PEG recovered the cellular protein content reduced by SIM in proliferating myoblasts (*p* < 0.001). GGOH worsened the protein content that had been diminished by SIM in differentiated myotubes (*p* < 0.01). The effect of GGOH was not affected by CQ administration (*p* > 0.05); *** *p* < 0.001, for comparison between the means. Statistically significant differences from untreated control cells are marked by # (at least at the level of *p* < 0.05, ns—non significant). The results are indicative of three independent experiments performed in eight replicates.

Overall cellular protein levels measured by SRB incorporation increased at day 3 and 5 upon CQ or Chol-PEG administration in comparison to untreated control. Statins had the opposite effect, causing a marked drop in the protein content which, in turn, was reversed by concomitant GGOH administration in proliferating myoblasts but not in differentiating or differentiated myotubes (Figure 2a,b). Combined treatment with GGOH in SIM-treated cultures led to a significant drop in the protein content in differentiated myotubes (Figure 2b).

**Figure 3:**
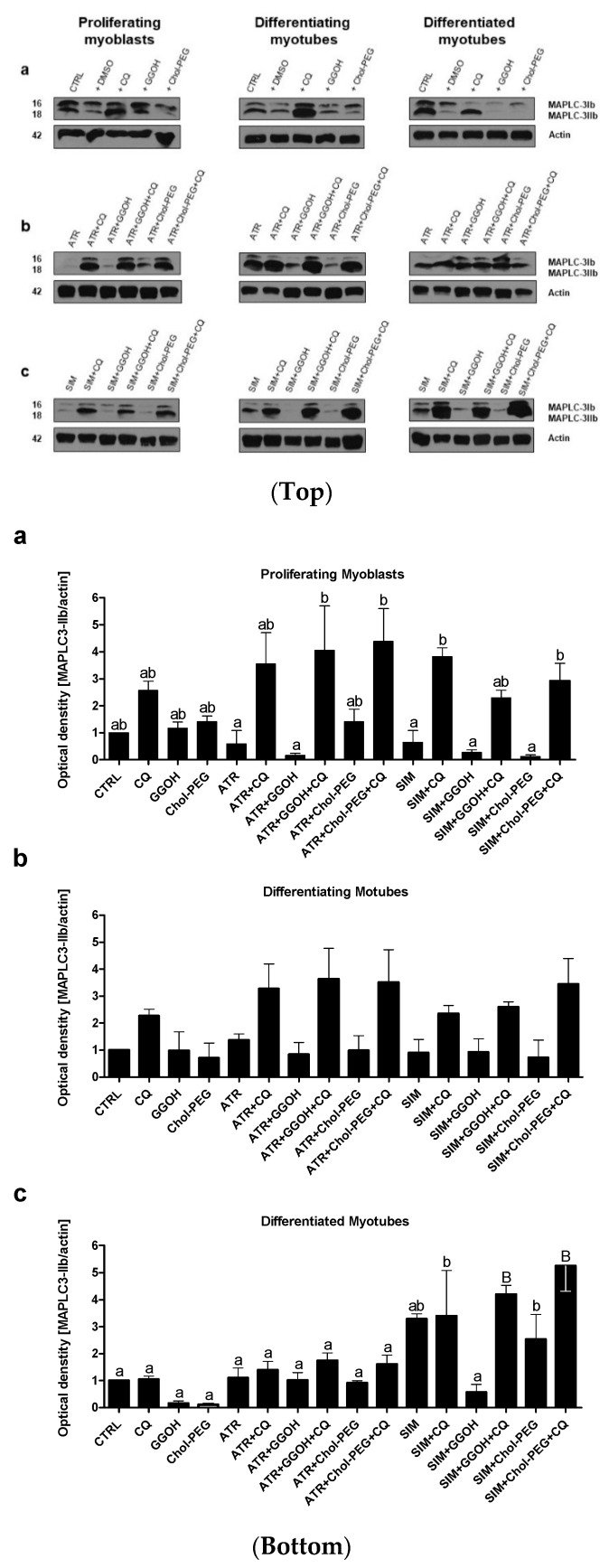
**Top.** Autophagy flux. Western blots of MAPLC-Ib/IIb with regard to actin. Effect of geranylgeraniol (GGOH, 10 µM) on the protein expression levels affected by MEV pathway modulators (atorvastatin—ATR, simvastatin—SIM) or water-soluble cholesterol (Chol-PEG) in the presence or absence of chloroquine (CQ, 10 mM). (**a**) In untreated myoblasts, the expression levels of MAPLC3-Ib/IIb protein increased accordingly with the differentiation stage. MAPLC3-Ib/IIb expression levels increased considerably upon CQ treatment. (**b**) ATR markedly reduced MAPLC3-Ib/IIb protein expression levels in proliferating myoblasts but differentiating and differentiated myotubes. Irrespective of co-treatment, CQ administration elevated MAPLC3-Ib/IIb protein expression levels equally, with no differences observed between the treatments. (**c**) SIM markedly reduced MAPLC3-Ib/IIb protein expression levels in proliferating myoblasts but not differentiating and differentiated myotubes. Irrespective of co-treatment, CQ administration elevated MAPLC3-Ib/IIb protein expression levels equally, with no differences observed between the treatments. Representative blots. The results are indicative of three independent experiments. **Bottom**. Autophagy flux. Densitometry analysis of MAPL3-IIb protein expression levels calculated in relation to house-keeping protein (actin). The effect of non-sterol isoprenoid, GGOH, and soluble cholesterol treatments on MAP LC3-IIb in C2C12 myoblasts affected by statins (atorvastatin—ATR; simvastatin—SIM) or water-soluble cholesterol (Chol-PEG). Differentiating C2C12 myoblasts exposed for 24, 72, or 120 h to statins (IC_50_), (Day 1—proliferating myoblasts; Day 3—differentiating myotubes; Day 5—differentiated myotubes). Different small letters indicate statistical significance (*p* < 0.05), whereas different capital letters represent values with high statistically significant (*p* < 0.01) differences between the means, respectively. The results are indicative of three independent experiments.

With regard to autophagy, progressive accumulation of the MAPLC-3IIb on day 1 and 3 but not at day 5 of the differentiation program was observed in C2C12 cells (Figure 3a). The use of statins barely affects MAP LC3IIb at any differentiation step (Figure 3b,c, *p* > 0.05). Thus, statin-dependent myotoxicity does not evoke a large adaptive response via accelerated autophagy in muscle cells. Neither GGOH nor water-soluble cholesterol (Chol-PEG) affected MAPLC-3IIb protein expression levels in statin-treated cells during CQ-induced autophagy inhibition (Figure 3b,c, *p* > 0.05). These data indicate that autophagy is central for myogenic differentiation (particularly at day 3) but neither GGOH- nor Chol-PEG could release muscle cells from statin-induced myotoxicity. It cannot be explained by augmented autophagy in muscle cells.

### 3.2. Pan-Caspase (Z-VAD-FMK) Inhibitor Could Partially Retain Muscle Cell Viability Reduced by Statins in Differentiating and differentiated myotubes but Not in Proliferating Myoblasts

**Figure 4:**
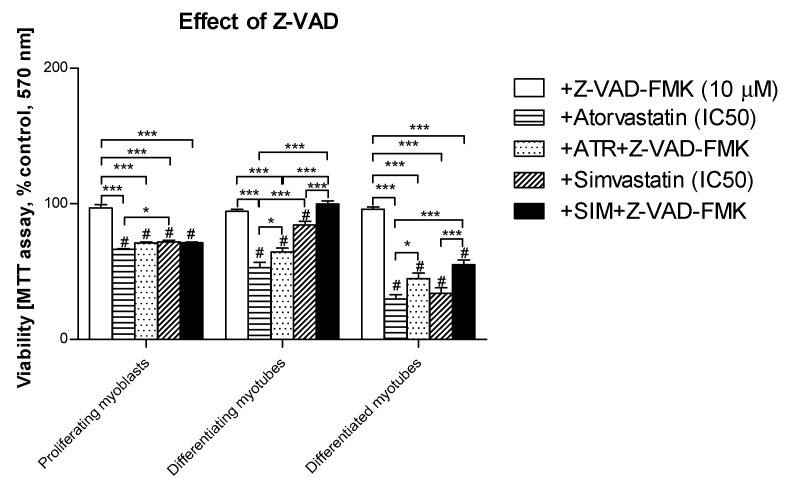
Effect of pan-caspase inhibitor Z-VAD-FMK on the cell viability (MTT assay) affected by MEV pathway modulators (atorvastatin—ATR, simvastatin—SIM). Differentiating C2C12 myoblasts were exposed for 24, 72, or 120 h to statins (IC_50_), (Day 1—proliferating myoblasts; Day 3—differentiating myotubes; Day 5—differentiated myotubes). ATR diminished the fraction of viable cells (IC_50_). Z-VAD-FMK itself did not affect cell viability, moreover, it could not recover ATR- or SIM-treated proliferating myoblasts (*p* > 0.05), but it significantly increased viable differentiating and differentiated myotubes treated with ATR or SIM (*p* < 0.05 and *p* < 0.001, respectively). * *p* < 0.05, *** *p* < 0.001, for comparison between the means. Statistically significant differences for untreated control cells are marked by # (at least at the level of *p* < 0.05). The results are indicative of three independent experiments performed in eight replicates.

The remaining Z-VAD-FMK pan-caspase inhibitor did not affect the cell viability at the concentration used (10 μM). When Z-VAD-FMK was administered to differentiating C2C12 cells, the protective effect of the pan-caspase inhibitor against statin-dependent myotoxicity was hardly observed at day 1, but it significantly protected cells at day 3 and 5 of myogenesis (Figure 4). These data suggest that apoptosis is involved in statin-induced muscle cell death during muscle cell fusion and further increases the muscle syncytium to fully grown myotubes.

### 3.3. Increased Magnitude of Autophagy in Differentiating Skeletal Muscle Cells Is Inversely Proportional to Procaspase-3 Cleavage

**Figure 5:**
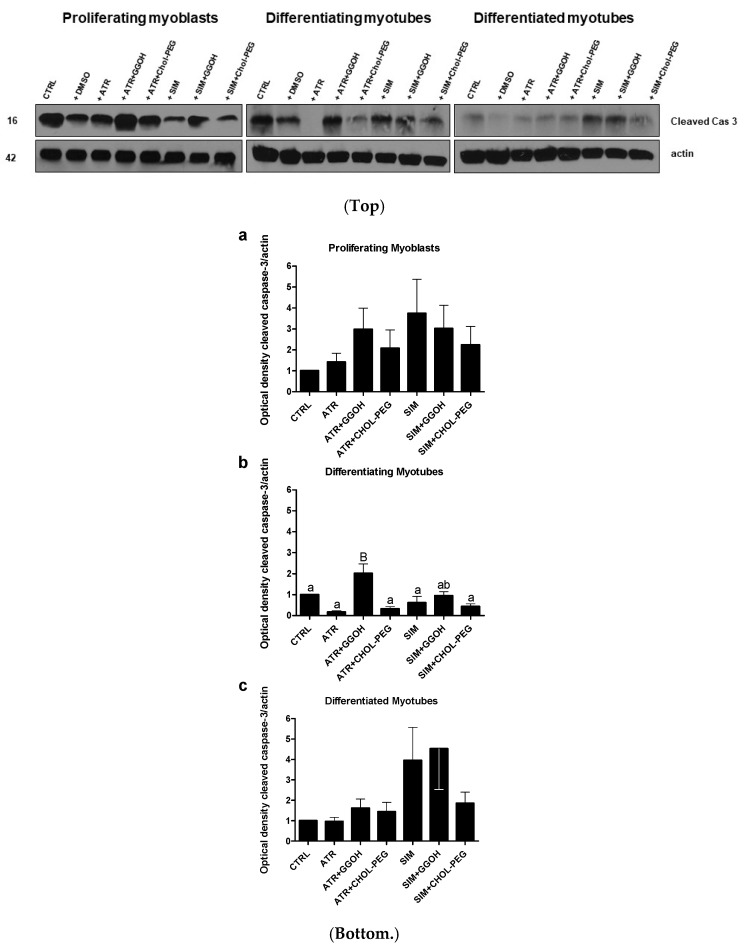
**Top.** Western blots of cleaved caspase-3 with regard to actin. Effect of geranylgeraniol (GGOH, 10 µM) on cleaved caspase-3 protein expression levels affected by MEV pathway modulators (atorvastatin—ATR, simvastatin—SIM) or water-soluble cholesterol (Chol-PEG). In untreated myoblasts, the expression levels of cleaved caspase-3 protein (cleaved cas 3) decreased accordingly with the differentiation stage. ATR and SIM markedly reduced cleaved cas 3 protein expression levels in proliferating and differentiating but not in differentiated myotubes. Irrespective of co-treatment, GGOH administration elevated cleaved cas 3 protein expression levels and, again, without observing the differences found in differentiated myotubes. In contrast to GGOH, the Chol-PEG co-treatment did not affect cleaved cas 3 protein expression levels regardless of co-treatment. Representative blots. The results are indicative of three independent experiments. **Bottom.** Densitometry analysis of cleaved caspase-3 (cleaved cas 3) protein expressions levels calculated in relation to house-keeping protein (actin). The effect of non-sterol isoprenoid GGOH and soluble cholesterol treatments on cleaved cas 3 in C2C12 myoblasts affected by statins (atorvastatin—ATR, simvastatin—SIM) or water-soluble cholesterol (Chol-PEG). Differentiating C2C12 myoblasts exposed for 24, 72, or 120 h to statins (IC_50_), ((**a**) Day 1—proliferating myoblasts; (**b**) Day 3—differentiating myotubes; (**c**) Day 5—differentiated myotubes). Different small letters indicate statistically significant (*p* < 0.05), whereas different capital letters indicate statistically highly significant (*p* < 0.01) differences between the means, respectively. The results are indicative of three independent experiments.

When autophagy was stimulated during myogenesis, the cleavage of caspase-3 progressively diminished in the following days (Figure 5, *p* < 0.05). The decreasing expression levels of the cleaved caspase-3 protein are in striking contrast to the evident MAPLC3-IIb accumulation in differentiating and differentiated myotubes (Figure 3b,c). Thus, the aforementioned observations point to an inverse relationship between autophagy and apoptosis that is well known in the literature. Moreover, the recovery of viable muscle cells from statin-induced myotoxicity upon GGOH or Chol-PEG administration could not be fully explained by autophagy induction. Interestingly, in differentiating myotubes (extensive fusion of myoblasts), protein expression levels of the cleaved caspase-3 were elevated by co-treatment with ATR or GGOH (Figure 5b, *p* < 0.001).

### 3.4. Inhibition of Calpains (μ- And m-) But Not Z-VAD-FMK Mimics the Effect of GGOH on Statin-Induced Myotoxicity

**Figure 6:**
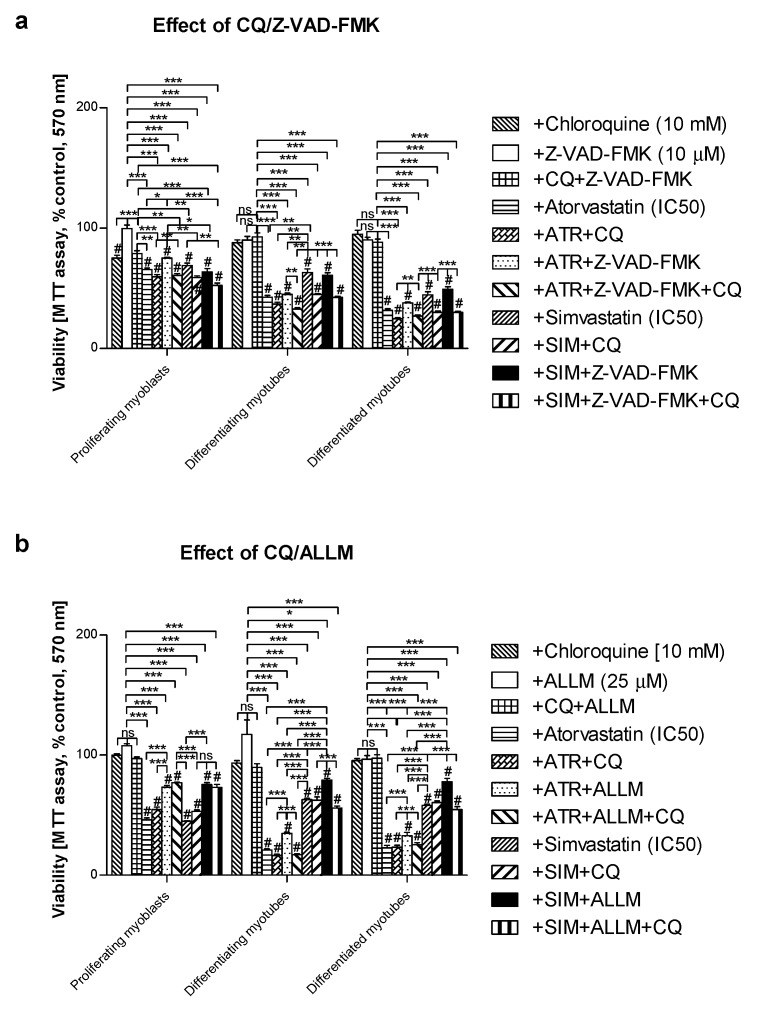
Effect of pan-caspase inhibitor Z-VAD-FMK or calpain inhibitor ALLM on cell viability (MTT assay) affected by MEV pathway modulators (atorvastatin—ATR, simvastatin—SIM) in the presence or absence of chloroquine (CQ, 10 mM). Differentiating C2C12 myoblasts were exposed for 24, 72, or 120 h to statins (IC_50_), (Day 1—proliferating myoblasts; Day 3—differentiating myotubes; Day 5—differentiated myotubes). (**a**) Both ATR and SIM diminished cell viability (IC_50_). Z-VAD-FMK itself had no effect on cell viability (*p* > 0.05). However, it overruled the myotoxicity evoked by ATR but not SIM administration in proliferating myoblasts only (*p* < 0.05). Upon CQ addition, the cell viability that had already been reduced by ATR or SIM co-treatment shrunk considerably further (*p* < 0.01). (**b**) ALLM itself had no effect on cell viability (*p* > 0.05). In contrast to Z-VAD-FMK, ALLM (25 μM) markedly recovered cells from ATR- or SIM-dependent myotoxicity in proliferating myoblasts, differentiating and differentiated myotubes (*p* < 0.001). ALLM recovered muscle cell viability less efficiently in ATR- than in SIM-dependent SAM. Statistically significant differences from untreated control cells are marked by # (at least at the level of *p* < 0.05, ns—non significant). * *p* < 0.05, ** *p* < 0.01, *** *p* < 0.001. The results are indicative of three independent experiments performed in eight replicates.

In order to determine whether statins induce caspase-dependent or caspase-independent cell death in C2C12 cells, the pan-caspase inhibitor Z-VAD-FMK vs. μ- and m-calpain inhibitor ALLM were administered individually with or without statins and/or CQ (Figure 6). As mentioned previously, Z-VAD-FMK (10 Μm) could not fully recover muscle cells from statin-induced myotoxicity (Figure 4, Figure 6a). On the contrary, ALLM at 25 μM (the highest concentration used did not affect the cell viability) efficiently blocked statin-induced myotoxic effects (*p* < 0.001, Figure 6b). These data suggest that statins may affect skeletal muscle cells through the caspase-independent mechanism, probably through calpain protease activation triggered by the Ca^2+^ signal. Calpains were previously reported to induce Ca^2+^-dependent while antioxidant inhibited muscle cell death [42]. Furthermore, Ca^2+^ flux in the mitochondrial matrix is thought to control the cell viability.

### 3.5. The Effect of GGOH or Chol-PEG co-Treatment Upon Mitochondrial Cytochrome C Oxidase Protein Expression Levels in Statin-Treated C2C12 Muscle Cells

**Figure 7:**
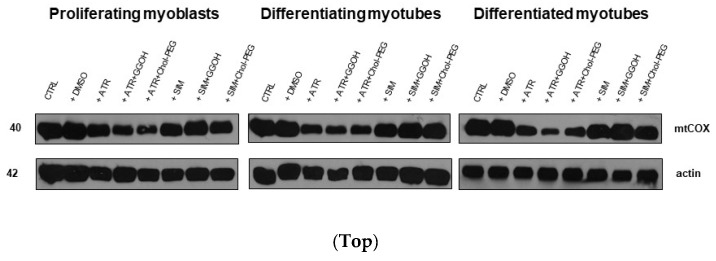

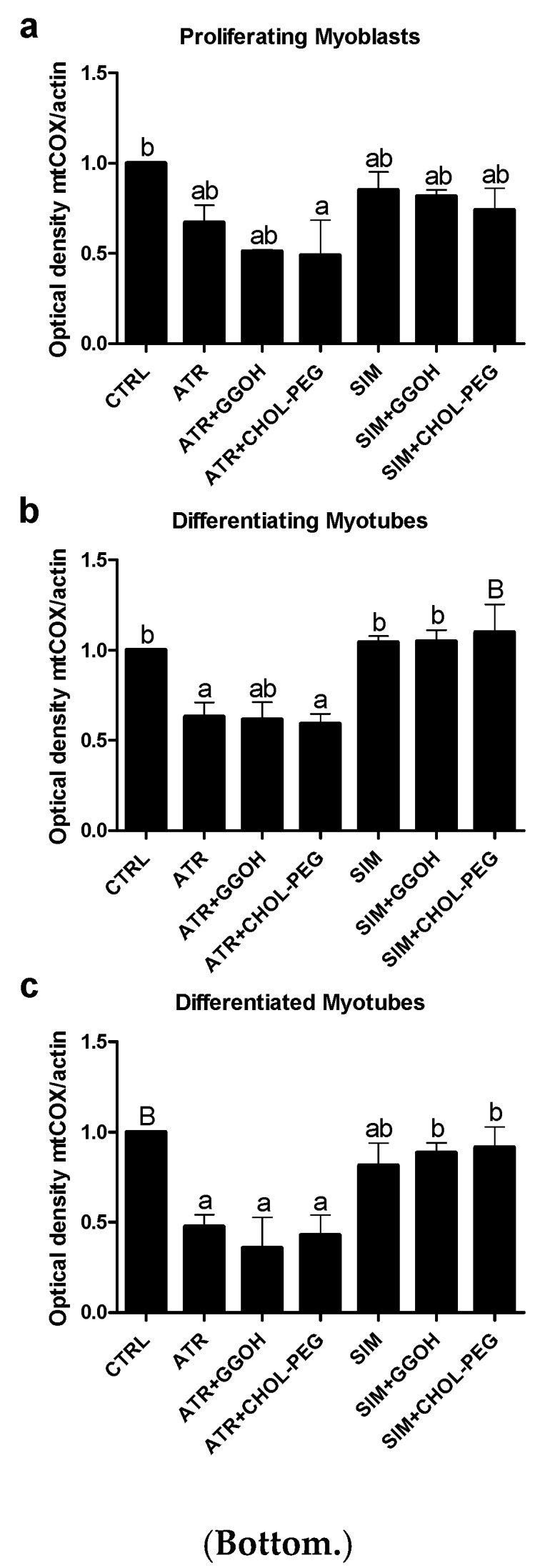
**Top.** The effect of GGOH or Chol-PEG co-treatment upon mitochondrial cytochrome c oxidase protein expression levels in statin-treated C2C12 muscle cells. In untreated myoblasts, the expression levels of cytochrome c oxidase (COX) protein remained high throughout muscle differentiation. COX expression levels dropped considerably upon ATR treatment. ATR markedly reduced COX protein expression levels in proliferating myoblasts and differentiating and differentiated myotubes. Irrespective of co-treatment with GGOH or Chol-PEG protein, expression levels of COX remained low in proliferating myoblasts and differentiating and differentiated myotubes. There was no such effect observed upon SIM treatment, whereby COX expression levels did not change either to SIM, GGOH or Chol-PEG co-treatment. Representative blots. The results are indicative of three independent experiments. **Bottom.** Densitometry analysis of mitochondrial cytochrome *c* oxidase subunit I (mtCOX1) protein expressions levels calculated in relation to house-keeping protein (actin). Effect of non-sterol isoprenoid GGOH and soluble cholesterol treatments on mtCOX1 in C2C12 myoblasts affected by statins (atorvastatin—ATR, simvastatin—SIM) or water-soluble cholesterol (Chol-PEG). Differentiating C2C12 myoblasts exposed for 24, 72, or 120 h to statins (IC_50_), ((**a**) Day 1—proliferating myoblasts; (**b**) Day 3—differentiating myotubes; (**c**) Day 5—differentiated myotubes). Different small letters indicate statistical significance (*p* < 0.05), whereas different capital letters indicate high statistically significant (*p* < 0.01) differences between the means, respectively. The results are indicative of three independent experiments.

Mitochondrial activity is measured in terms of cellular oxidative respiration determined by the cytochrome c oxidase (COX, EC 1.9.3.10) that reduces molecular oxygen (O_2_). This enzyme forms the respiratory complex IV, where electrons from the electron transport chain (ETC) together with some protons (1:1) lead to the synthesis of water molecules (2H_2_O), allowing maintenance of the ETC and proton gradient across the inner mitochondrial membrane. The subunit I of COX (mtCOI) is specifically encoded by mtDNA, and the evaluation of its expression was chosen to assess a statin-dependent mitochondrial impairment. From immunoblotting, it is obvious that ATR but not SIM reduced the expression levels of the mtCOI protein (Figure 7, *p* < 0.05–0.01). Neither GGOH nor Chol-PEG could undo the ATR-dependent decrease in mtCOI protein expression levels (Figure 7, *p* > 0.05). These results led to the important conclusion that, at the mitochondrial level, ATR-induced myotoxicity differs from SIM-dependent cell loss.

### 3.6. Mitochondrial Respiration in Statin-Treated C2C12 Muscle Cells. The Effect of GGOH and Chol-PEG Co-Treatment

**Figure 8:**
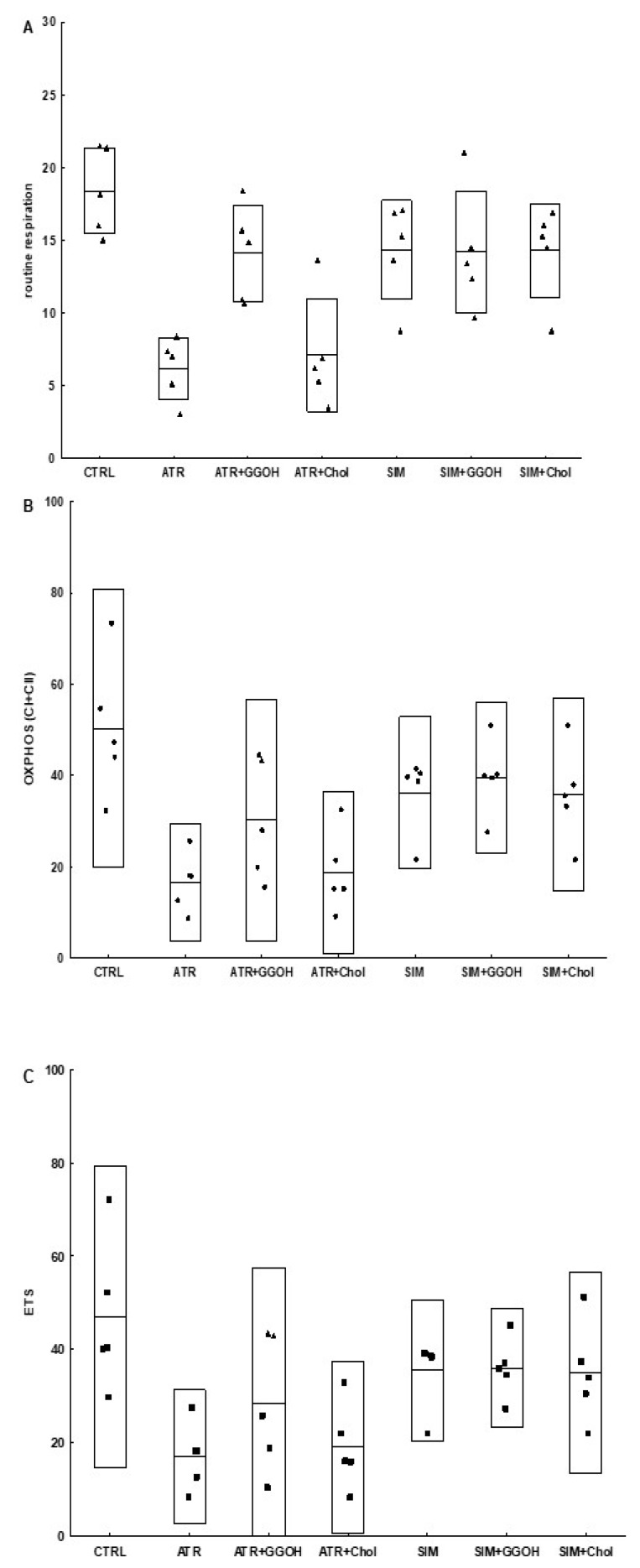
Selected parameters (routine, OXPHOS and electron transport system (ETS)) of mitochondrial respiration in permeabilized C2C12 myoblasts after treatment with statins at IC_50_ (ATR, atorvastatin; SIM, simvastatin) and geranylgeraniol (GGOH; 10 µM) or cholesterol (Chol-PEG; 1 mM). Data were expressed as the mean ± standard deviation (SD) and the raw data, *n* = 5. The significance of differences was estimated based on: One-way ANOVA test and Tukey’s test as post hoc; (**A**) *p* < 0.05 for ATR vs. ATR + GGOH, ATR vs. SIM, ATR + GGOH vs. ATR + Chol; *p* < 0.001 for CTRL vs. ATR; CTRL vs. ATR + Chol; (**B**) *p* < 0,001 for CTRL vs. ATR, *p* < 0,05 for CTRL vs. ATR + Chol; (**C**) *p* < 0,01 for CTRL vs. ATR, CTRL vs. ATR+Chol; more experimental details see ‘Material and Methods’.

In order to verify the suggestions that statins are harmful to skeletal muscle because of impaired mitochondrial bioenergetics, the experiments with mitochondria respirometry were carried out with oxygraph-2k Oroboros (see Materials and Methods section for details). Mitochondria were studied over the course of both individual statin treatments (ATR or SIM) and/or GGOH and Chol-PEG treatments. As shown in Figure 8 and Table 1, the ATR basal level of respiration (routine respiration) was decreased as well as, in the presence of substrates for mitochondrial complex I and II, OXPHOS I or OXPHOS I&II and ETS were impaired, confirming the negative effect of ATR on the activity of the oxidative phosphorylation (OXPHOS) and the electron transport system (ETS). While ATR impaired mitochondrial respiration, a completely different result was found in the case of SIM. No statistically significant changes in the mitochondrial respiration were observed for SIM when muscle cells were stimulated with ADP and/or FCCP compared to non-treated control cells. However, while the co-treatment with GGOH improved, water-soluble cholesterol (Chol-PEG) did not reverse the harmful effect of ATR on mitochondrial respiration. Thus, GGOH restored the mitochondrial function monitored in ATR-treated cells. GGOH and Chol-PEG given individually did not affect mitochondria respiration.

### 3.7. Viability of C2C12 Muscle Cells during Autophagic Flux. The Cytoprotective Effect of GGOH Is Fully Dependent on Geranylgeraniol Transferase I (GGT-I) and Could Be Reversed by Inhibition of Autophagy with Chloroquine

**Figure 9:**
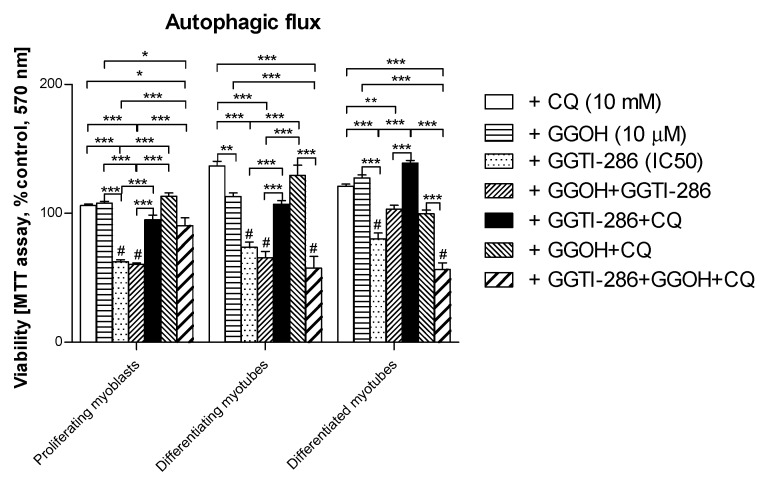
The effect of geranylgeraniol (GGOH, 10 µM) on cell viability (MTT assay) affected by geranylgeranyl transferase I (GGTI-286, IC_50_) in the presence or absence of chloroquine (CQ, 10 mM). Differentiating C2C12 myoblasts were exposed for 24, 72, or 120 h to GGTI-286 (IC_50_), (Day 1—proliferating myoblasts; Day 3—differentiating myotubes; Day 5—differentiated myotubes). Neither CQ nor GGOH affected the cell viability in comparison to control untreated cells (*p* > 0.05); GGOH could not rescue the muscle cell viability reduced by GGTI-286 in proliferating myoblasts and differentiating and differentiated myotubes (*p* > 0.05). The effect of GGOH was not affected by CQ administration (*p* > 0.05); CQ effectively reversed the negative effect of GGTI-286 on cell viability. GGOH blocked the effect of CQ on the GGTI-286-dependent drop in cell viability in differentiating and differentiated myotubes (*p* < 0.001) but not in proliferating myoblasts (*p >* 0.05). * *p* < 0.05, ** *p* < 0.01, *** *p* < 0.001, for comparison between the means. Statistically significant differences from untreated control cells are marked by # (at least at the level of *p* < 0.05). The results are indicative of three independent experiments performed in eight replicates.

Autophagic flux was employed to determine whether the cytoprotective effect of GGOH is linked to autophagy activation. As mentioned above, autophagy could not fully explain the GGOH-induced myoprotective effect. Furthermore, chloroquine (CQ) used to inhibit autophagic flux did not impair cell viability either (Figure 9, *p* > 0.05). Administration of GGTI-286, a specific inhibitor of geranylgeranyl-transferase type I (GGT-I, EC 2.5.1.59), reduced the cell viability by almost half (*p* < 0.001) and the additional use of GGOH remained fruitless. This observation confirms our previous study [33] where a similar relationship between GGT-I and GGOH was reported. Furthermore, the addition of CQ fully reversed the GGTI-286-dependent loss in the muscle cell viability, whereas concomitant use of GGOH worsened the effect of CQ (Figure 9, *p* > 0.05).

## 4. Discussion

The incidence of SAM (in an era when statins are widely used and known to be efficient drugs in the treatment and prophylaxis of atherosclerosis), and its complications which mainly affect the cardiovascular system, require a detailed explanation, new measures and modalities. Finally, even though statins induce lethal rhabdomyolysis in less than 0.1% of cases, the issue is urgent as statins are the most popular medication in the worldwide treatment of cholesterolemia. Our former observations and other reports showed that sparing non-sterol isoprenoids prevent SAM [19,33,43,44]. Moreover, FOH and GGOH (probably through the “salvage pathway”) restore the muscle cell viability that was reduced by ATR or SIM [34]. Thus, in the circumstances described above, non-sterol isoprenoids are important for skeletal muscle integrity and function. There is indirect evidence that isoprenes, rather than cholesterol, control muscle function. Patients with inherited metabolic diseases associated with impaired activity of enzymes in cholesterologenesis, such as desmosterolosis [43] or lathosterolosis [45], do not experience myopathy in the set of clinical symptoms. In turn, mevalonic aciduria caused by mevalonate kinase deficiency with a resultant drop in isoprenes is linked to skeletal myopathy [46]. In addition, a blockade of either squalene synthase or squalene epoxidase downstream of FPP, which is a common substrate for DOH, GGOH and cholesterol, is not associated with myotoxicity [15,16]. It appears that pathophysiology that explains the relationship between statin administration and statin toxicity in skeletal muscle due to a low level of non-sterol isoprenoids does apply for neuromuscular toxicity. However, a bulk of evidence points to the cytoskeleton and the small GTPases as fundamental cellular components that determine cell viability. It is isoprenylation, rather than the cholesterol–protein interaction and isoprenylation of target protein(s) such as G-proteins, that require close examination. The latter is finely regulated by guanosine nucleotide exchange factors (GEFs) and GTPase-activating proteins (GAPs) that regulate gene expression, thin and thick filaments, their spatial organization, as well as vesicular transport along the microtubular network [47,48]. Disturbances in actin and the microtubular network with resultant inhibition of vesicular transport are frequently observed at the ultrastructural level upon statin use in “in vitro” experiments [49,50]. Widespread vacuolization detected by us and others at the ultrastructural level in statin-treated neuronal and muscle cells was initially assigned to autophagy activation. However, we could not find convincing evidence except for an elevated number of autophagic vacuoles [33,51]. It was assumed that if autophagy is to be blamed, the present study must exploit “autophagy flux” with the aim to solve the uncertainty. To do this, studies were conducted in identical experimental conditions with chloroquine administered to block the degradation phase of autophagy. It was demonstrated that autophagy inhibition elevated MAPLC-3IIb protein expression levels in untreated muscle cells (control). However, it was hard to reveal any significant difference between treatment with GGOH alone and GGOH combined with statins (Figure 3). Although we showed that Beclin-1 protein expression levels rose in response to GGOH [33], in the present work, we could not find any effect of GGOH on the expression levels of MAPLC3-IIb during autophagic flux. As it is known, the MAPLC3-IIb expression levels rather than p62 protein (sequestome) limit autophagy (autophagosome formation). Sequestome determines a specific substrate for MAPLC3-IIb and it is unlikely to widen the scope (p62 could be considered as cargo protein for selective autophagy) [52,53]. Altogether, unaltered MAPLC3-IIb protein expression levels sufficiently proved that autophagy was hardly stimulated by GGOH. 

The “in vitro” model of myogenesis from C2C12 mouse myoblasts seems to be a suitable approach to study substances that can powerfully prevent statin-induced myotoxicity. Statins kill muscle cells by an unidentified molecular mechanism, sometimes described as apoptosis [16,19,27,54] and sometimes as necrosis [44,55], in conjunction with the activation of caspases and/or calpains as the administration of pan-caspase inhibitor Z-VAD-FMK or calpain inhibitor ALLM which reversed the myotoxicity of statins (*p* < 0.05–0.001, Figure 2 and Figure 4). Previously, bonds between the mevalonate pathway and programmed cell death (PCD) and/or autophagy were ascertained during studies in patients with mevalonate kinase deficiency described in the review reports [56,57]. Again, the application of exogenous isoprenoids overruled the deregulation of the mevalonate pathway at the level of protein isoprenylation [58]. Surprisingly, in our experiment, it was not caspase-3 which could account for the apoptotic effect of statins, because we could not demonstrate an increase in the cleavage of this protease by ATR or SIM treatments (Figure 5, *p* > 0.05). Moreover, the cytoprotective effect of GGOH was connected with elevated cleavage of caspase-3, suggesting this cysteine-dependent aspartate-directed protease was involved in muscle cell survival rather than the execution of PCD. Upon cytoskeletal disruption, caspase-3-independent PCD can proceed through caspase-1, caspase-2 [59] or calpain cascade [42].

Notably, in this and a previous study, we demonstrated that SAM can be prevented by co-treatment with GGOH in C2C12 muscle cells (*p* < 0.001, Figure 1, Figure 2). This non-sterol isoprenoid was shown to control Rap1 protein prenylation through geranylgeranyltransferase I (GGTase-I) [33]. Other isoprenoids were noticeably less effective (FOH) whereas Chol-PEG had no effect. GGOH was thus selected for further study due to its potent cytoprotective effect. We tested the hypothesis that GGOH augments autophagy and that autophagy is a tool in the maintenance of a viable population of muscle cells. A series of “autophagy flux” experiments were performed where CQ inhibited the terminal step in autophagy (degradation phase) in examined muscle cells. Irrespective of the differentiation state (proliferating myoblasts, differentiating, or differentiated myotubes), autophagy inhibition did not reveal any significant differences in the expression levels of MAPLC3-IIb between the treatments vs. non-treated control. Therefore, we conclude that autophagy cannot give an explanation for GGOH-dependent protection of muscle cells from SAM, whereas it should be considered as an indispensable process for the proper differentiation of skeletal muscle.

Mitochondria are often indicated as potential targets of statins. This belief was substantiated by the testimony of lower expression levels of mitochondrial enzymes included in aerobic respiration and disturbed Ca^2+^ homeostasis [21,23,24,25,26,27]. In fact, ATR markedly depleted muscle cells from COX subunit I (mtCOI), confirming the mitochondrial contribution to ATR-dependent myotoxicity (Figure 7, *p* < 0.05–0.01). This is reflected in the results of measurements of mitochondrial bioenergetics and is consistent with the reports of the stronger negative effect of less lipophilic statins on these organelles. In studies using an oxygraph-2k Oroboros instrument, we have shown that ATR impairs complex I and II of the mitochondrial respiratory chain, and GGOH co-treatment reverses this effect. However, we did not observe an increase in the expression of mtCOI upon the addition of GGOH to ATR-treated cells. This may indicate that the ATR primarily affects complex I and II, and the GGOH protective effect also applies only to the mentioned respiratory chain complexes.

The cytoprotective effect of GGOH was subject to additional scrutiny with regard to cell viability. As could be expected from the previous study [33], GGOH prevented muscle cell viability through geranylgeranyl transferase I activity (GGT-I, EC 2.5.1.59) as inhibition of the enzyme with GGTI-286 completely blocked the GGOH cytoprotective effect (Figure 9, *p* > 0.05). Surprisingly, the effect of GGTI-286 was fully reversed by CQ, indicating that autophagy is the negative regulator of protein prenylation. Thus, autophagy can effectively counteract the process of protein prenylation with GGT-I, whereby prenylated proteins (i.e., RAP1) are no longer capable of sustaining muscle cell viability. Eventually, the inhibition of autophagy with CQ was confirmed by altered MAPLC3-IIb expression. The molecular mechanism of autophagy-dependent removal of prenylated proteins requires further examination.

In this study, we demonstrated the central role played by GGOH in the protection of muscle cell viability reduced by SAM in an in vitro model of myogenesis. ATR did not, but SIM made use of caspases in diminishing cell viability as the pan-caspase inhibitor Z-VAD-FMK significantly recovered muscle cells from SIM-dependent cell loss. Lower viability of muscle cells was, however, associated with caspase-3 independent muscle cell death. The decrease in viability of ATR and SIM is dependent on calpains in proliferating myoblasts. However, it becomes independent in SIM-induced SAM in differentiating and differentiated myotubes. Similarly, the opposite effect of ATR vs. SIM was observed in mitochondria; ATR was found to be toxic to mitochondria whereas SIM was not.

## 5. Conclusions

(i) Autophagy is essential for myogenesis.
(ii) The myoprotective effect of GGOH is not dependent on autophagy activation.
(iii) Atorvastatin does, whereas simvastatin does not impair mitochondrial bioenergetics.
(iv) GGOH prevents muscle cell viability in SAM through the inhibition of calpains rather than caspases.

## Figures and Tables

**Table 1:** Selected respiratory states calculated for C2C12 myotubes.

Compound	OXPHOS I	CII
CTRL	26.91 ± 8.01	34.08 ± 11.09
ATR	7.28 ± 3.36	9.24 ± 6.22
ATR + GGOH	20.35 ± 5.23	20.15 ± 11.57
ATR + Chol-PEG	9.20 ± 5.13	10.61 ± 4.69
SIM	19.65 ± 3.74	23.46 ± 7.89
SIM + GGOH	22.81 ± 5.00	18.23 ± 6.05
SIM + Chol-PEG	19.79 ± 6.58	22.32 ± 6.83

Data were shown as mean ± SD, *n* = 5. All values of the oxygen consumption by mitochondria of C2C12 cells were corrected by ROX. Oxygen consumption was expressed as pmol O2 per second per 1 million cells. The significance of differences was estimated based on: One-way ANOVA test and Tukey’s test as post hoc; (OXPHOS I) *p* < 0.05 for CTRL vs. ATR + GGOH, ATR vs. SIM, ATR + GGOH vs. ATR + Chol; *p* < 0.005 for CTRL vs. ATR + Chol; *p* < 0.001 for CTRL vs. ATR; (C II) *p* < 0.01 for CTRL vs. ATR, CTRL vs. ATR + Chol; for more experimental details see ‘Material and Methods’. Legend: OXPHOS I (ADP-stimulated respiration supported Complex I), C II (mitochondrial respiration after Complex I inhibition, in the presence of succinate addition).

## Supplementary Materials

The following are available online at https://www.mdpi.com/2077-0383/8/5/694/s1. Figure S1: In vitro visualization of mitochondrial morphology with MitoTracker Green (excitation/emission 490/516) on Day 1, 3 and 5.
