# Supplementary material for: Diverse Action of Selected Statins on Skeletal Muscle Cells—An Attempt to Explain the Protective Effect of Geranylgeraniol (GGOH) in Statin-Associated Myopathy (SAM)
Source: J Clin Med. 2019 May 16;8(5):694. doi: 10.3390/jcm8050694 (PMC6572681; doi:10.3390/jcm8050694)
Supplement: Supplementary file 1 [file jcm-08-00694-s001.pdf]

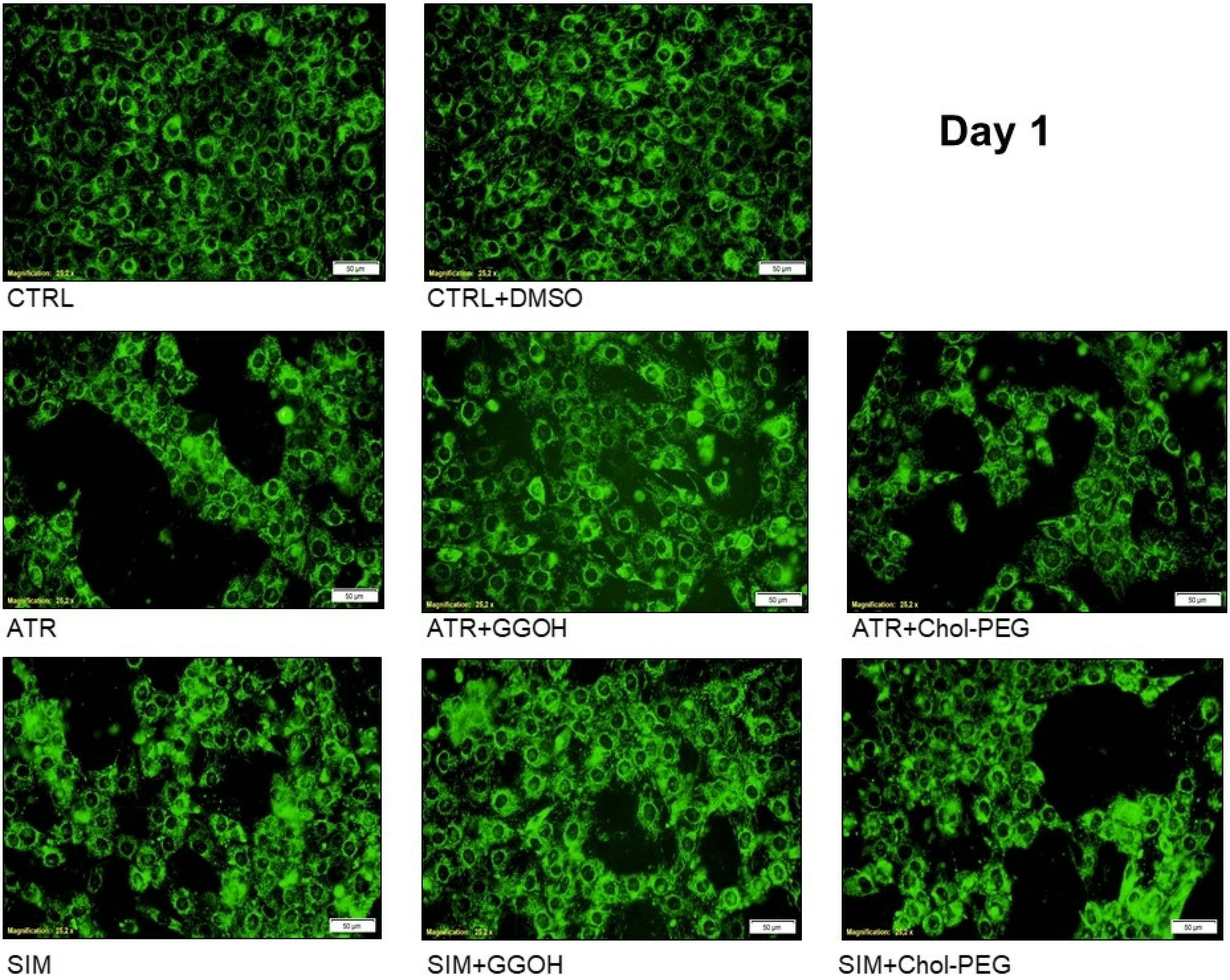
Day 1
CTRL
CTRL+DMSO
ATR
ATR+GGOH
ATR+Chol-PEG
SIM
SIM+GGOH
SIM+Chol-PEG

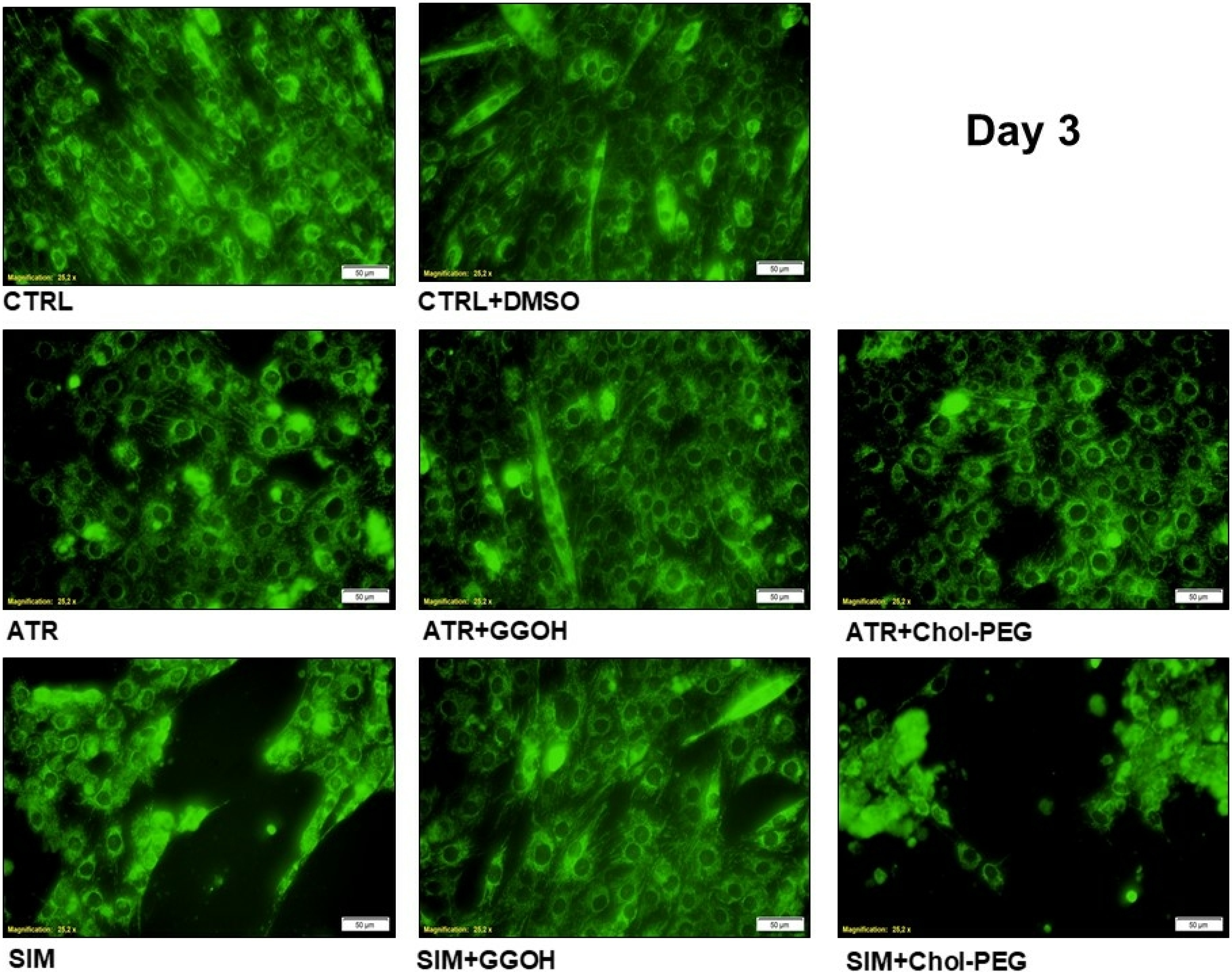
Day 3
CTRL
CTRL+DMSO
ATR
ATR+GGOH
ATR+Chol-PEG
SIM
SIM+GGOH
SIM+Chol-PEG

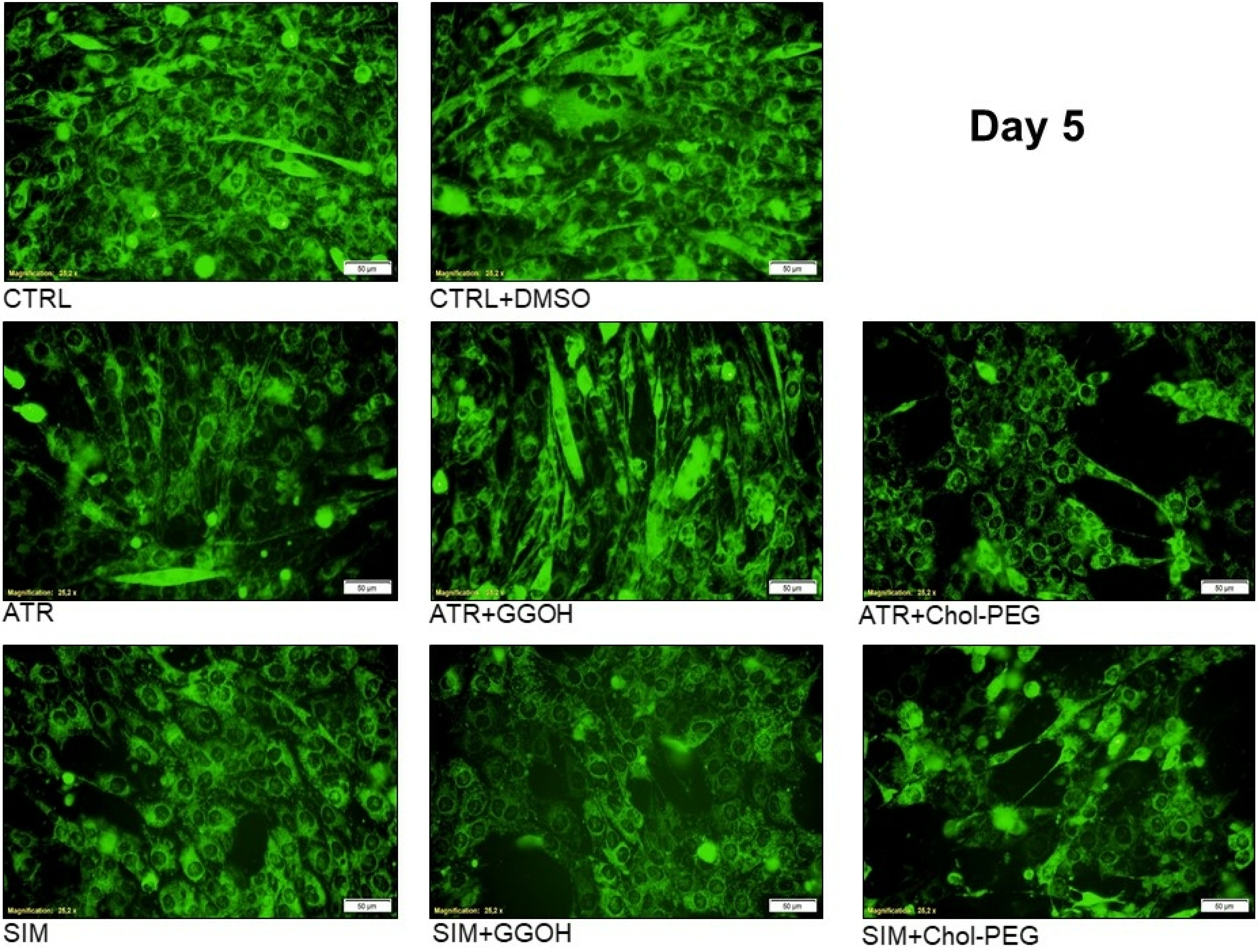
Day 5
CTRL
CTRL+DMSO
ATR
ATR+GGOH
ATR+Chol-PEG
SIM
SIM+GGOH
SIM+Chol-PEG
